# Development of a readiness model for industry 4.0 using Analytical Hierarchy process and fuzzy inference system: Bangladesh perspective

**DOI:** 10.1016/j.heliyon.2023.e23664

**Published:** 2023-12-12

**Authors:** S.M. Fahim Faisal, Sajal Chandra Banik, Pranta Sen Gupta

**Affiliations:** a Department of Mechatronics and Industrial Engineering, Chittagong University of Engineering and Technology, Bangladesh; b Department of Mechanical Engineering, Chittagong University of Engineering and Technology, Bangladesh

**Keywords:** Analytical hierarchy process, Fuzzy logic, Industrial revolution, Industry 4.0, Fuzzy inference system

## Abstract

The rapid acceleration of innovation and the marvels of science and technology have introduced mankind to a new era known as the Fourth Industrial Revolution or Industry 4.0. As a developing country, Bangladesh should not lag behind in the concept of the domain known as Industry 4.0. A substantial number of industries across Bangladesh are coming forward to take on the challenges associated with this novel concept. It is, however, reported that many of them are now at the embryonic stage and only taking pilot initiatives. Before entering the domain of Industry 4.0, an industry must check its readiness and maturity to access the field, and that's where it comes to the importance of the readiness model. The main objective of this research is to propose a new readiness model based on the perspective of Bangladesh and developing countries like Bangladesh. Analytical Hierarchy Process and Fuzzy Inference System has been used for developing the model. There are four readiness levels in the proposed model that dictate the readiness of any particular industry. Through systematic literature review and expert opinion, 6 dimensions and their associated field have been identified for the determination of readiness level. In the later part of the research, a case study on one of the companies in the garments industry has been accomplished, and a radar chart is presented in order to illustrate the readiness of that garments industry. The results from the case study indicate that the ABC garments industry is in the Survival stage and several recommendations are then provided to cope better in that situation.

## 1 Introduction

**Fig. 1:**
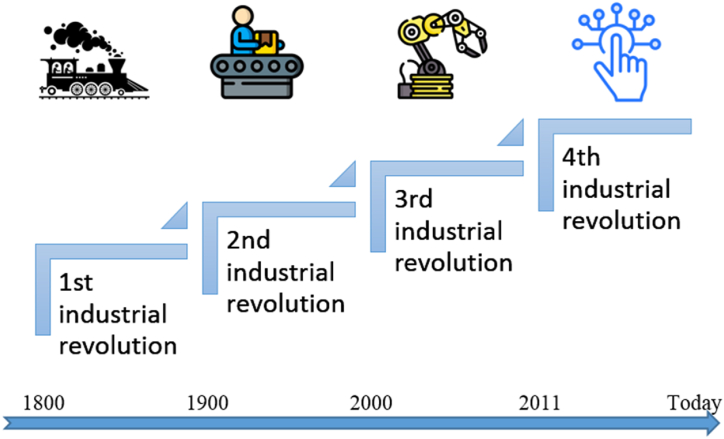
Industrial revolutions throughout history.

Industrialization is the foundation upon which the economic growth of a country depends. From the dawn of civilization until today, industries have seen significant advances in technical and technological aspects. For centuries, most of the goods were manufactured by hand or by utilizing the labor of animals. This scenario was changed at the end of the 18th century with the introduction of manufacturing processes [1]. The first revolution was production process mechanization, which produced steam and water through the use of power [2]. With the discovery of electricity, mankind embarked on the second industrial revolution. The invention of electricity allowed mass production to take place. Then, at the beginning of 1969, the third industrial revolution started to take place, which can be expressed and characterized by the use of automation in industry [1,3]. The use of computers and programmable controllers started to take place, and supply chains became more complex and larger. The industrialization process, as a consequence, became a phenomenon around the world. This eventually led us to the concept of "Industry 4.0″ or "Fourth Industrial Revolution" [4]. The term “Industry 4.0” is now not merely a buzzword; rather, almost all the developed countries are now participating in this revolution. Along with the developed countries, the countries in Southeast Asia are also entering the domain of Industry 4.0. Since Bangladesh is a developing country, she should also consider stepping into the terrain of Industry 4.0, and the initiation of the process is ongoing. Fig. 1 represents industrial revolutions throughout the history of mankind.

A few authors have provided a more comprehensive framework for developing a national plan for Bangladesh to take part in the global Fourth Industrial Revolution (4IR) [5]. The adoption of Industry 4.0 (I4), which entails integrating cutting-edge technology to revolutionize industries and increase productivity, has been developing steadily in Bangladesh [6]. Several initiatives have been launched to assist with this implementation. The Government of Bangladesh has demonstrated a dedication to Industry 4.0 as well as a commitment to digital transformation. To encourage the adoption and creation of new technologies, they have created the ICT Division and numerous policy frameworks. Automation, robotics, and IoT (Internet of Things) have made considerable advancements in Bangladesh's manufacturing industry [7]. Bangladesh is making investments to modernize its infrastructure, which will improve digital connection and increase the availability of broadband internet. This offers a solid basis for implementing Industry 4.0. Research and development organizations are actively working on cutting-edge technologies including cloud computing, blockchain, big data analytics, and artificial intelligence (AI) [8]. These advancements contribute to the overall Industry 4.0 landscape in Bangladesh.

According to a number of researchers, Industry 4.0 comprises a wide range of components. Most of the writers and researchers stated CPS (Cyber-physical system) as the main feature and component of Industry 4.0. It is stated as the adaptive technologies to manage interconnected systems between their physical resources and computing capacities [9]. CPS refers to the integration of physical systems (such as machinery, devices, and sensors) with digital technologies and connectivity, forming a networked infrastructure [10]. Real-time data sharing, communication, and collaboration between physical items and computer systems are made possible by this integration. CPS is essential to Industry 4.0's goal of streamlining and modernizing industrial processes. It enables improved automation, monitoring, and control of production processes, which increases productivity, flexibility, and efficiency. The use of CPS can help an industry make better decisions, do predictive maintenance, optimize its resources, and be more responsive to customer needs [11]. Additionally, CPS permits the concept of a "digital twin," which is a virtual version of a real product or process for simulation and analysis. Jay Lee et al. [12] have developed a 5C architecture that describes every stage of a cyber-physical system. In this regard, Moreno et al. [13] propose the construction of a digital twin for a sheet metal punching machine, with the conclusion that there is a necessity for machining process virtualization of this kind, which improves the manufacturing process. This empowers the development of smart factories.

To enter the era of Industry 4.0, each industry must assess its readiness in the context of this topic. For this, maturity models regarding Industry 4.0 are used to check the readiness and maturity of the industry. The work presented in this paper is the formulation of a readiness model with a view to checking the readiness of an industry in the Industry 4.0 domain.

The remaining portion of the paper is structured as follows: In section 2, previous literature reviews are comprehensively analyzed in Section 3 provides an overview of the proposed model's framework. The completed case study and the outcomes of applying the methodology are shown in Section 4. Section 5 concludes by summarizing the findings.

## 2 Literature review

The definition of maturity varied among authors and in various contexts. The definition of the term "Maturity" is quite important in formulating the maturity model in any aspect. Nikkhou [14] refers to the term maturity as a state of being in an absolutely perfect condition. Maturity can be used as an assessment criterion and defined as total, ideal, or ready, according to Proença and Borbinha [15]. It might serve as a symbol for moving from an elementary stage to the more advanced final stage. A maturity model, according to Tarhan et al. [16], describes the optimal realistic progression for systems in a wide range of business fields that require distinct stages of maturity. Furthermore, maturity models are described as a useful tool for evaluating processes or organizations from multiple viewpoints [15]. Backlund et al. [17] have mentioned that maturity models seem to become increasingly relevant for evaluating organizations. Nikkhou et al. characterized maturity models as a method for describing ideal development to the desired transition by using a few step-by-step phases or stages [14]. Maturity models allow organizations to review and standardize evaluation outcomes, being more toward a target level, and analyze organizational elements such as capabilities, vulnerabilities, and advantages by sequencing maturity levels from basic to developed: Initialized, Managed, Specified, Quantitatively Controlled, and Optimized [6]. In the paper, the distinction between readiness and maturity models is clarified as follows: readiness models determine whether or not an enterprise is ready to begin planning and implementation; however, maturity models attempt to explain which maturity stage an enterprise is at.

According to a literature survey on maturity models conducted by Gökalp et al. [18], the model is a structure that assists enterprises in achieving their most critical goals by offering detailed guidelines and a strategic blueprint. According to the report, there has been an increase in Industry 4.0 research in recent years. Still, there is a research gap due to limited research on the use of maturity models, and the majority of the models available do not help manufacturing organizations structure a holistic perspective. There are also no models based on a well-defined system that includes practices, all the inputs, and all the outputs. As a result, there is still a need for a standardized Industry 4.0 readiness and maturity model.

The disparity between readiness and maturity models, according to Schumacher et al., is dependent on the time being considered. The readiness model, in particular, evaluates the system before the transition, while the maturity model evaluates the system by capturing the as-it-is condition in progress. In other words, the assessment is done during the change [19]. Schumacher et al. maturity model released in 2016, seems to provide the existing state of strategies implemented by various manufacturing industries and evaluate the effectiveness of Industry 4.0 strategies from nine different perspectives: product (digitalization of the product), operations (decentralization of processes), customer (digitalization of sales/services), strategy (implementation of the I4 roadmap), leadership (management competencies and methods), technology (utilization of mobile devices), governance (regulations for I4), culture (knowledge sharing), and people (autonomy of employees) [19].

Rajbhandari et al. had done a case study in Nepal [20] on the framework of Industry 4.0, which is a relatively new area of research in the context of Nepal. The goal of that study was to investigate whether a given variable genuinely influences industrial preparedness. SPSS and AMOS were used to analyze the collected data. According to the survey, the industrial sector's shortage of skilled workers is the main obstacle to Industry 4.0 adoption. Structured Equation Modeling (SEM) was employed to assess the hypothesis put forth in the literature review.

Chinese manufacturing enterprises are found to have significant enthusiasm and expectations for Industry 4.0, but just 57 % of them are completely prepared for I4.0 innovations, according to a McKinsey poll of 130 Chinese firm officials. According to this survey, these figures for China are significantly lower than those for the United States (71 %) and Germany (68 %). The main cause in a number of manufacturing sectors is an inadequate understanding of the value of these technologies. Technologies associated with I4.0 are manufacturing-information technology integration with an intricate and interconnected architecture [21]. Standard assessment makes it challenging to determine the effect of these technologies; nevertheless, extra evaluation for sustainability advantages and preparedness may increase their strategic adoption, even if it adds to the complexity of the process [22]. As a result, Dalenogare et al. [23] and Raut et al. [24] both cite this as a crucial and unresolved area of study in the assessment of Industry 4.0 and the preparedness of the industry.

With the creation of the Industry 4.0 maturity model based on fuzzy rules, Caiado et al. [25] attempted to remove human subjectivity from the evaluation procedure. They added Monte Carlo simulation and fuzzy logic to the Industry 4.0 self-assessment tool, which they then used in a case study to validate. They defined the dimensions (the assessment criteria) through thorough literature research and then used interviews and focus groups to create the model.

A unique readiness factor computation approach based on decision support systems and process planning was introduced by Trstenjak et al. [26]. By utilizing decision support systems and statistical techniques built into the model, the model makes it possible to define the best possible strategic plan for digitization while minimizing the impact of human subjectivity and quantifying qualitative criteria. They conducted a case study at a Croatian metal machining firm to collect and validate the findings.

Babi et al.'s [27] rating system of businesses according to their industrial level of maturity makes use of the TOPSIS and AHP decision support methodologies. The significance of things in three key categories—technique, organization, and personnel—has been assessed by a qualified panel of 38 CEOs in Croatia. The components were weighted using AHP, and the relative proximity index of the business to Industry 4.0 was then determined using the TOPSIS approach.

A "specific tool to assess enterprises concerning manufacturing digitalization" was created by Pirola et al. [28] and is known as the "Digital Readiness Level 4.0″ model (DRL 4.0). The results offer recommendations for future development in addition to the preparedness level. The model was created using a multi-case research methodology. The average readiness score is computed after an interview.

Abdul-Hamid et al. [29] conducted research that seeks to examine the drivers of I4 in the Circular economy (CE) and the linkage between the two through an ecological theory of modernization viewpoint in the Malaysian palm oil sector by taking into account the significance of I4 in the CE. In this research, the drivers of Industry 4.0 in the CE are identified using the Delphi approach and the fuzzy-DEMATEL method to create a diagram of cause and effect for the Malaysian palm oil sector.

The results showed that the model could assist the companies in order to provide a self-assessment of their degree of Industry 4.0 implementation. In this paper, the readiness model for Bangladesh perspective is based on Becker's step-by-step process for developing the maturity model [30].

## 3 Framework of the proposed readiness model

Following Becker's step-by-step process for the development of the maturity model, the concept of the model is derived from the following works-

• IMPULS – Industrie 4.0 Readiness [31].
• Industry 4.0/Digital Operations Self-Assessment [32].
• A maturity model for Industry 4.0 Readiness [19].
• SIMMI 4.0 [33,34].
• The Connected Enterprise Maturity Model [35].
• Readiness for adoption of Industry 4.0 in Nepal [20].
• Industry 4.0 Readiness Calculation—Transitional Strategy [26].

**Fig. 2:**
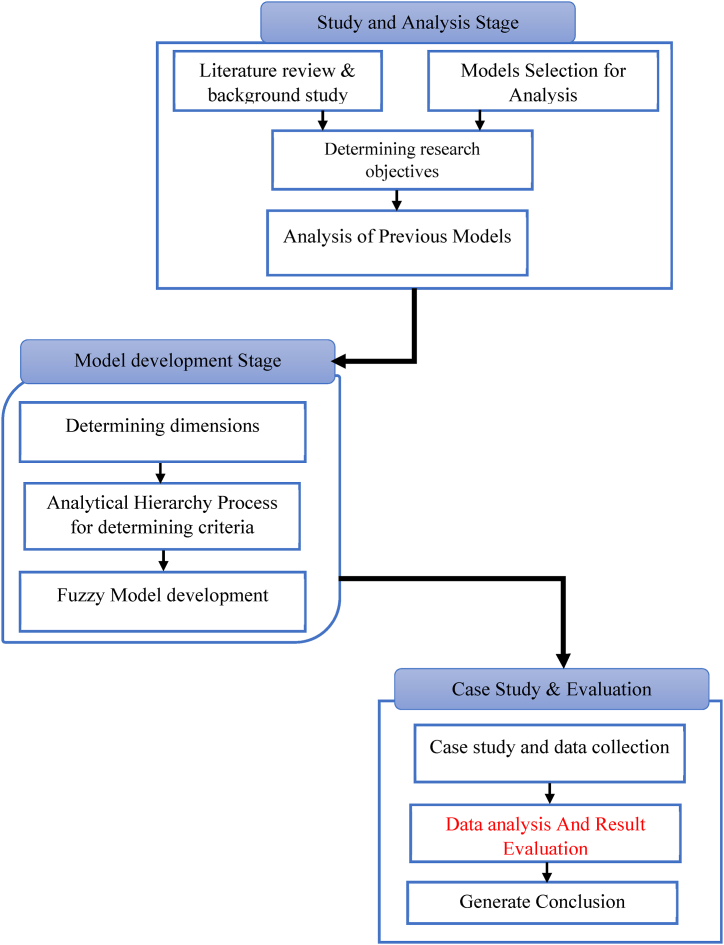
Flow chart of research work.

In Fig. 2, a procedural framework of the research work is illustrated.

Some of the previous research regarding maturity and readiness model formulation for Industry 4.0 is now summarized in Table 1.

**Table 1:** Several research regarding maturity and readiness model formulation for Industry 4.0

Model/Research Name	Research Context	Maturity Levels	Dimensions	Used Methodology
IMPULS – Industrie 4.0 Readiness [31]	Industry 4.0 readiness	6 maturity levels	6 dimensions	Graphical Analysis
Industry 4.0/Digital Operations Self-Assessment [32]	Digital readiness for Industry 4.0	3 maturity levels	6 dimensions	Survey Research
A maturity model for Industry 4.0 Readiness [19]	Industry 4.0 maturity	Likert-scale maturity levels (1–4)	9 dimensions	Questionnaire Survey
SIMMI 4.0 [33,34]	Industry 4.0 maturity	5 maturity stages	3 dimensions	Not Defined
The Connected Enterprise Maturity Model [35]	IT readiness	5 maturity stages	4 dimensions	Metric Analysis
Readiness for adoption of Industry 4.0 in Nepal [20]	Industrial readiness index	5 readiness states	7 dimensions	SPSS and AMOS
Industry 4.0 Readiness Calculation—Transitional Strategy [26]	Decision support systems	5 readiness states	5 dimensions	Analytical Hierarchy Method and Statistical Analysis
Readiness Model using AHP & FIS (Presented/proposed model)	Industry 4.0 readiness	4 readiness states	6 dimensions	Analytical Hierarchy Process and Fuzzy Inference System

### 3.1 Determination of Model's maturity levels & dimensions

In this part of the research, the maturity levels and dimensions are needed to set up. By systematic review of the other maturity models, the dimensions and maturity levels are selected. The model will help almost all manufacturing industries; therefore, the dimensions and the readiness model are made keeping that in mind. This model will mainly help developing countries like Bangladesh, so this fact was also considered while selecting the model's maturity levels and dimensions. As Bangladesh and those countries that do not yet have industry4.0 available, the maturity levels will work for them as readiness levels in most cases.

There are four readiness levels in the proposed model. The four levels are now given below-

Level 0: Outsiders.

Level 1: Novice.

Level 2: Survival.

Level 3: Professional.

The descriptions are given below for each level are now given.

Level 0: Outsiders.

It defines a company standard that does not follow any of the Industry 4.0 standards. Any of the criteria are shockingly low and kind of totally absent in most of the scenarios.

Level 1: Novice.

A stage of readiness in which the organization has initiated some of the steps and has some pilot initiatives at various sectors and divisions. Modern technologies might not be at full implementation, and they are not yet ready to transform themselves.

Level 2: Survival.

This readiness level indicates a readiness level where the company has great initiatives in its functional departments. They have a good automation level along with a planned business process. They are in a good and balanced state but need some strategy formulated and implemented to develop their readiness.

Level 3: Professionals.

The firm's products are characterized as intelligent and data-driven services, indicating a high degree of sophistication—enterprise market level in terms of convergence, information sharing, and flexibility.

The dimensions are selected along with the associated fields. A tabular form of the dimensions with the associated fields is now given in Table 2.

**Table 2:** Dimensions, associated Fields and items of the proposed model.

Dimensions	Associated Fields And Items
Management & Leadership	The willingness of entrepreneurs, management methods
Planning & Strategy	Investment, innovation, the vision of Industry 4.0 roadmap.
Smart Products& Machines	Digitalization of products, automation level in the industry, data collection and analytics of data, product tracking
Business Process	Business models, marketing process, digital process & cloud usage.
Employee & Training	Skill development, acquisition, skill training of employees.
Customers Feedback	Customer's data utilization, feedback method.

### 3.2 Analytical hierarchy process for criteria weights

**Fig. 3:**
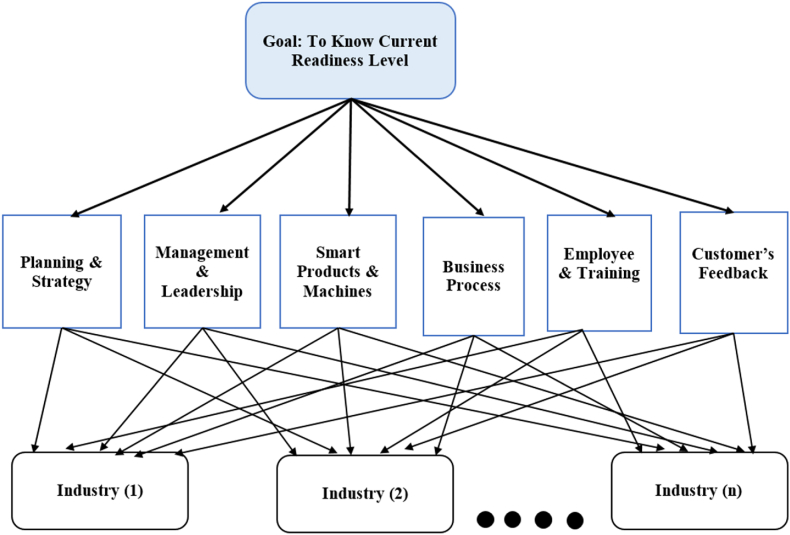
Hierarchical structure of the AHP model.

In this part of the research work, it is needed to determine the weights of the dimensions or criteria used in the proposed readiness model. As all the dimensions are not of equal importance, assigning weights to all these dimensions is required. Only using expert opinion seems like a good way to assign weights to the criteria. Still, the Analytical Hierarchy Process (AHP) is also a proven and useful method to assign weights to criteria in the multi-criteria decision-making process. The six dimensions that are chosen act as the criteria in the AHP, and different types of industry are the alternatives in this case. Fig. 3 indicates the whole AHP in a single picture with the goal, criteria, and all the alternatives.

The first step in making the AHP model is to define the goal, criteria, and all the possible alternatives, which was done in the previous step. Then some steps have to be followed to determine the criteria weights needed for the research work. The first and most important step is the formation of a pair-wise comparison matrix.

The pair-wise comparison matrix is formed by taking all the dimensions into account. Saaty [36] defined this relative importance for pair-wise comparison, which is given in Table 3. For the formulation of our pair-wise comparison matrix, this relative importance scale is used.

**Table 3:** Relative importance scale of pair-wise comparison [36].

Relative Importance	Definition
1	Equal importance
3	Moderate importance
5	Strong importance
7	Very Strong importance
2,4,6	Intermediate Values(For example, 2 for equal to moderate importance)

In this research, the pairwise comparison matrix is developed using the data obtained from the expert panel. The expert panel consists of 11 academicians and 14 high-ranking officials, like CEOs, heads and managers of several highly reputed industries all across Bangladesh. Their opinion and dataset form the pair-wise comparison matrix which prioritize the dimensions accordingly.

After that, all the dimensions are written as D1, D2, and so on for the presentation purpose in the pair-wise comparison matrix. All the dimensions are defined and designated as.

D1 = Management & Leadership.

D2 = Planning & Strategy.

D3 = Smart Products & Machines.

D4 = Business Process.

D5 = Employee & Training.

D6 = Customer's Feedback.

Then the pair-wise comparison matrix M is made using all the designated dimensions D1 to D6. The pair-wise comparison matrix M is made based on expert opinion and a systematic literature review. The matrix M is now given below.

$$M=\left[\begin{array}{c} \begin{array}{cccccc} \;D1 & D2 & D3 & D4 & D5 & D6 \end{array}\\ \begin{array}{c} D1\\ D2\\ D3\\ D4\\ D5\\ D6 \end{array}\;\begin{array}{cccccc} 1 & 1/2 & 2 & 3 & 2 & 3\\ 2 & 1 & 2 & 3 & 2 & 4\\ 1/2 & 1/2 & 1 & 2 & 2 & 3\\ 1/3 & 1/3 & 1/2 & 1 & 2 & 3\\ 1/2 & 1/2 & 1/2 & 1/2 & 1 & 2\\ 1/3 & 1/4 & 1/3 & 1/3 & 1/2 & 1 \end{array} \end{array}\right]$$

The calculations that are needed for the AHP model are carried out in Table 4, Table 5, Table 6. Firstly, the pair-wise comparison matrix is made using all the designated dimensions D1 to D6 and the sum is calculated which is given in Table 4.

**Table 4:** AHP calculation (pair-wise comparison matrix formation).

	D1	D2	D3	D4	D5	D6
**D1**	1	1/2	2	3	2	3
**D2**	2	1	2	3	2	4
**D3**	1/2	1/2	1	2	2	3
**D4**	1/3	1/3	1/2	1	2	3
**D5**	1/2	1/2	1/2	1/2	1	2
**D6**	1/3	1/4	1/3	1/3	1/2	1
**SUM**	4.66	3.08	6.33	9.83	9.5	16

**Table 5:** AHP calculation (determination of criteria weights).

	D1	D2	D3	D4	D5	D6	Criteria Weights
**D1**	0.214	1.162	0.316	0.305	0.2105	0.188	0.233
**D2**	0.42	0.324	0.315	0.305	0.211	0.25	0.303
**D3**	0.107	0.163	0.157	0.203	0.2105	0.1875	0.1714
**D4**	0.072	0.109	0.078	0.102	0.211	0.188	0.1265
**D5**	0.107	0.163	0.079	0.05	0.105	0.125	0.106
**D6**	0.072	0.081	0.052	0.034	0.053	0.063	0.06

**Table 6:** AHP calculation (for consistency checking).

	D1	D2	D3	D4	D5	D6	Sum	Criteria Weights
**D1**	0.233	0.152	0.343	0.379	0.212	0.18	1.49	0.233
**D2**	0.466	0.303	0.343	0.379	0.21	0.24	1.9	0.303
**D3**	0.1165	0.152	0.1714	0.253	0.212	0.18	1.08	0.1714
**D4**	0.077	0.10	0.086	0.1265	0.22	0.18	0.78	0.1265
**D5**	0.1165	0.152	0.086	0.063	0.106	0.12	0.64	0.106
**D6**	0.077	0.076	0.057	0.042	0.053	0.06	0.365	0.06

In the next step at Table 5, the matrix is normalized by dividing all the elements of the column by the sum of the columns, and then they are added to obtain the criteria weights that are needed for the model.

After determining the criteria weights, the consistency needs to be checked. This is done by calculating the Consistency Ratio (CR). Now, a matrix is developed by multiplying the criteria weights with all the elements of the pair-wise comparison matrix. Table 6 is required for the calculation of λ _max_, which will be later required for the calculation of consistency ratio.

The consistency index (CI) is a measure used in the Analytical Hierarchy Process (AHP) to assess the consistency of pairwise comparison judgments made by decision-makers.

In AHP, decision-makers compare different criteria or alternatives using a pairwise comparison matrix. These comparisons are then transformed into numerical values, which represent the relative importance or preference of one criterion or alternative over another. To ensure the reliability of the decision-making process, it is essential to check the consistency of these judgments. The CI is a quantitative measure that determines the extent of inconsistency in the pairwise comparison matrix. The CI is calculated by taking the difference between the maximum eigenvalue (λmax) and the number of criteria n divided by n-1.

The CI ranges from 0 to +∞. A CI value of 0 indicates perfect consistency, while higher values indicate increasing inconsistency. To determine whether the pairwise comparison judgments are acceptable, the CI is compared to a random consistency index (RI) value, which depends on the number of criteria being compared. If the CI value exceeds the RI value, it suggests that the judgments are inconsistent and require further adjustments.

A widely used measure called the consistency ratio (CR) is also calculated by dividing the CI value by the corresponding RI value which is given in equation (1).

(1) CR=CI/RI

The CR provides a more comprehensive assessment of consistency, considering the number of criteria being compared. Typically, a CR value of less than 0.10 is considered acceptable and indicates an acceptable level of consistency.

Overall, the consistency index and consistency ratio are important tools in the AHP methodology to ensure the validity and reliability of decision-making processes relying on pairwise comparisons.

Sum of all the elements and the criteria weights are mentioned in Table 6 for calculating λ _max_.

For all rows, we divide the sum by the criteria weights, $\frac{1.49}{0.233}$ = 6.39.

So, by doing this in each row, the values obtained are 6.39, 6.40, 6.30, 6.16, 6.05 and 6.08 respectively.

Then λ _max_ is calculated.

λmax=6.39+6.40+6.30+6.16+6.05+6.086

= 6.23.Now, the Consistency Index (CI) is calculated according to equation (2).

(2) $$\text{CI}=\frac{\lambda \;\max-n}{n-1}$$

$$=\frac{6.23-6}{6-1}$$

= 0.046.

Now, Consistency ratio (CR) is = $\frac{\text{Consistency}\;\text{Index}\left(\text{CI}\right)}{\text{Random}\;\text{Index}\;\left(\text{RI}\right)}$.

In Table 7, Random Index (RI) for n equals 1 to 6 is given, where n is the number of criteria or dimensions in this paper.

**Table 7:** Random index (RI) [37].

n	1	2	3	4	5	6
RI	0	0	0.58	0.90	1.12	1.24

So, Consistency ratio (CR) is = $\frac{\text{Consistency}\;\text{Index}\left(\text{CI}\right)}{\text{Random}\;\text{Index}\;\left(\text{RI}\right)}$.

$$=\frac{0.046}{1.24}$$

= 0.037.

As the Consistency Ratio (CR) is less than 0.10, pair-wise comparison matrix M is consistent and can be used for further evaluation.

### 3.3 Readiness determination using fuzzy logic

The final step of the research work is aimed at designing a fuzzy logic system in which companies or industries give their inputs as answers to the selected questionnaire. Then, after giving the input, it goes through a fuzzy inference system (FIS) and, through the defuzzification process, gives the output as the current readiness level of that selected industry.

Fuzzy logic is unique because it can categorize an object in more than one exclusive set where levels of truth or confidence are different [38].

In this research work, the Gaussian membership function is used to define all the inputs for making the fuzzy system. The inputs to the system are all the dimensions that were selected. The Gaussian membership function is usually represented as Gaussian (x: c, s) where c and s represent the mean and standard deviation. It is now shown in equation (3).

(3) $$\mu \left(x,c,s,m\right)=\exp\;[-\frac{1}{2}|\frac{x-c}{s}\hat{|}\left(m\right)\;]$$

MATLAB Fuzzy Logic Toolbox uses fuzzy logic principles for calculation. In this case, the input range [0, 1] is divided into four intervals [0.416, 0], [0.416, 0.333], [0.416, 0.667] and [0.1416, 1], which correspond to the membership levels "very low," "low," "medium," and "high," respectively.

For each interval, a membership value is assigned based on the degree of overlap between the input value and the interval. This is done using the Gaussian membership function with a standard deviation of 0.1416.

For example, if the input value is 0.1 and if it falls in a random interval of [0, 0.25]. The membership value for this interval can be calculated using the Gaussian membership function. The mean of the Gaussian function will be at the center of the interval, i.e., 0.125, and the standard deviation is given as 0.1416. By substituting these values into the Gaussian function, the membership value for this interval can be obtained.

Similarly, membership values are calculated for the other intervals based on the input value. These membership values are then used in subsequent fuzzy logic operations, such as fuzzy rule evaluation, aggregation, and defuzzification, to compute the final output or action to be taken based on the fuzzy logic system design.

For the output, the triangular membership function is used. Membership function μ(x) is defined in equation (4).

(4) $$\mu \left(x\right)=\left\{ \;\begin{array}{c} 0\;x\;<\;a\\ \frac{x-a}{x-b}\;a\leq x\leq b\;\\ \frac{d-x}{d-e}\;c\leq x\leq d\;\\ 0\;x\;>\;d \end{array}\right.$$

**Fig. 4:**
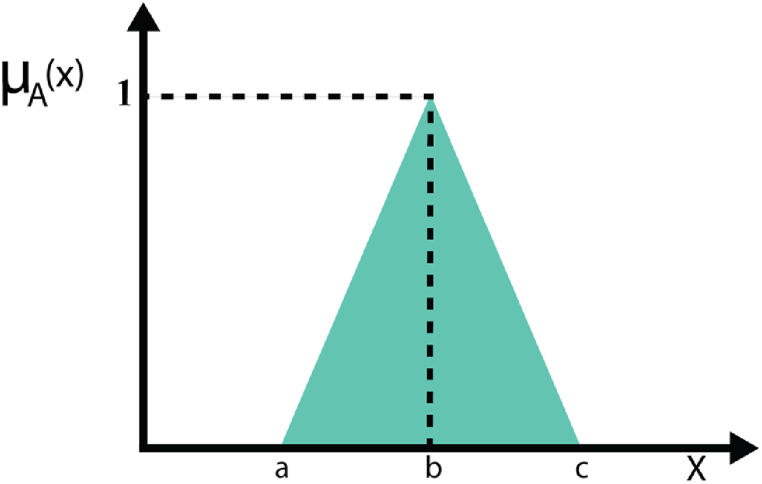
Triangular membership function.

In Fig. 4, the triangular membership function is now defined and illustrated.

MATLAB fuzzy logic toolbox is used for the fuzzy model development. The input to the fuzzy inference systems are as follows.

1. Management & Leadership
2. Planning & Strategy
3. Smart Products & Machines
4. Business Process
5. Employee & Training
6. Customer's Feedback

**Fig. 5:**
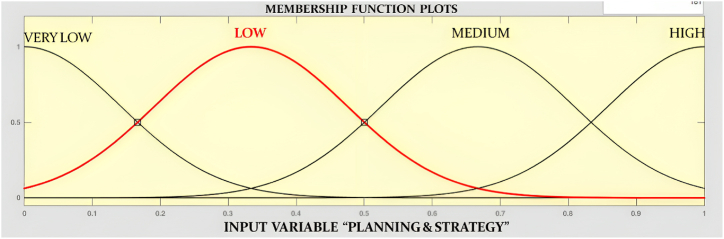
MATLAB fuzzy logic toolbox for FIS.

The output in the fuzzy logic toolbox is the current readiness level of the selected industry. Mamdani fuzzy inference system is used in the MATLAB fuzzy logic toolbox as it is the most established one. Fig. 5 indicates all the things that are done to formulate the fuzzy system in the fuzzy logic toolbox.

**Fig. 6:**
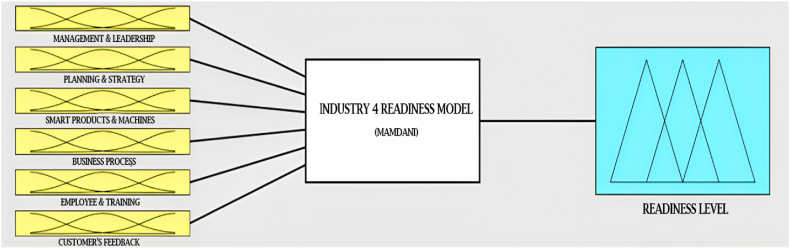
Planning & Strategy membership function.

Previously it was mentioned that the Gaussian membership function is used for all the inputs. Fig. 6 represents planning and strategy input in the fuzzy logic toolbox.

**Fig. 7:**
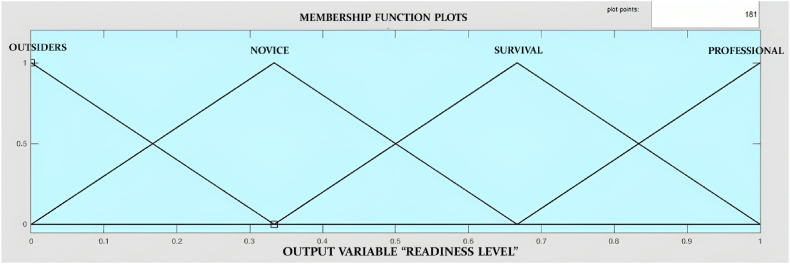
Readiness Level membership function.

Very low, low, medium, and high are used for all the input membership functions, and a standard deviation of 0.1416 is used in the Gaussian membership function. The output membership function is the readiness level of the selected industry, and for that, a triangular membership function is used. Fig. 7 represents the output membership function in the fuzzy logic toolbox of MATLAB.

For the formation of the fuzzy if-then rules, expert opinion and the criteria weights and ranking obtained from the Analytical Hierarchy Process (AHP) are used. The criteria weights that are found in the AHP process helped to rank the dimensions and give specific priority to different dimensions. In total, 25 rules are formulated for determining the readiness level of a particular industry.

In total, 25 rules are formulated for determining the readiness level of a particular industry, and Table 8 gives a brief description of all the rules that are used.

**Table 8:** Fuzzy if-then rules.

Management & Leadership	Planning & Strategy	Smart Products & Machines	Business Process	Employee & Training	Customer's Feedback	Readiness Level
Very Low	Very Low	Very Low	Very Low	Very Low	Very Low	Outsiders
Very Low	Low	Very Low	Low	Low	Low	Outsiders
Low	Very Low	Very Low	Low	Very Low	Low	Outsiders
Low	Very Low	Low	Low	Very Low	Low	Outsiders
Very Low	Low	Very Low	Very Low	Low	Very Low	Outsiders
Very Low	Low	Low	Low	Low	Very Low	Novice
Very Low	Low	Low	Low	Medium	Low	Novice
Medium	Low	Low	Very Low	Very Low	Very Low	Novice
Low	Medium	Very Low	Very Low	Very Low	Low	Novice
Low	Very Low	Medium	Medium	Low	Low	Novice
Low	Low	Low	Low	Low	Low	Novice
Low	Medium	Low	Low	Low	Medium	Survival
Medium	Medium	Low	Low	Low	Low	Survival
Low	Medium	Medium	Medium	Low	Low	Survival
Medium	Medium	Low	Low	Medium	Medium	Survival
Medium	Medium	Low	Medium	Low	Low	Survival

Management & Leadership	Planning & Strategy	Smart Products & Machines	Business Process	Employee & Training	Customer's Feedback	Readiness Level
High	High	Medium	High	Medium	High	Professionals
High	Medium	High	High	High	Medium	Professionals
Medium	High	High	Medium	High	High	Professionals
High	High	Medium	Medium	High	Medium	Professionals
High	High	High	High	High	High	Professionals
Low	Medium	Medium	Medium	Medium	Low	Survival
Medium	Medium	Medium	Medium	Medium	Medium	Survival
Medium	High	Medium	Medium	Medium	High	Survival
High	High	High	Medium	Medium	Medium	Professionals

## 4 Case study and results

According to Becker's model, validation of the model is required at the final data analysis stage. For that reason, a detailed case study is done in a single garment industry. The selected garments industry "X" is a sportswear-making company located in Cumilla, Bangladesh. Their main customers are buyers from many European countries. They make the jackets and trousers of many companies who have a great reputation around the world. Firstly, the questionnaire was asked to different top-level management personnel in the garments industry. For each dimension, 3 to 4 questions are asked. The questionnaire of smart products and machines dimension is now given below-

• What is the current level of automation in your industry?
• What is the procedure of the data collection technique?
• To what extent can products be tracked throughout their lifecycle? (The University of Warwick Maturity Model)

**Fig. 8:**
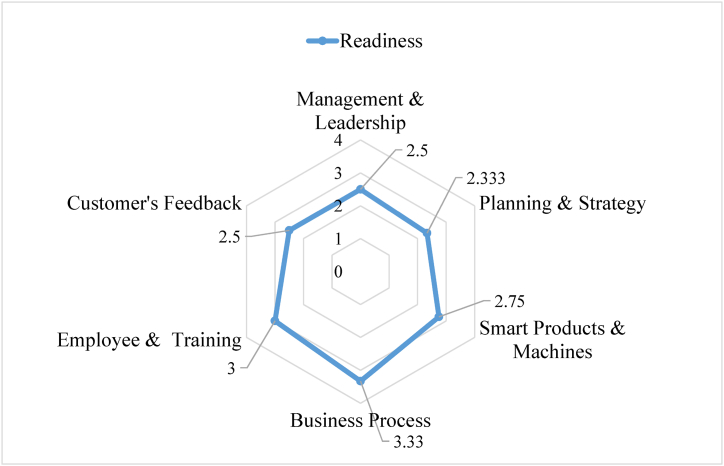
Readiness level of the garments industry (Radar Chart).

Then all the answers are taken, and then the readiness level of that garments industry is presented in a radar chart on a scale of four in Fig. 8.

**Fig. 9:**
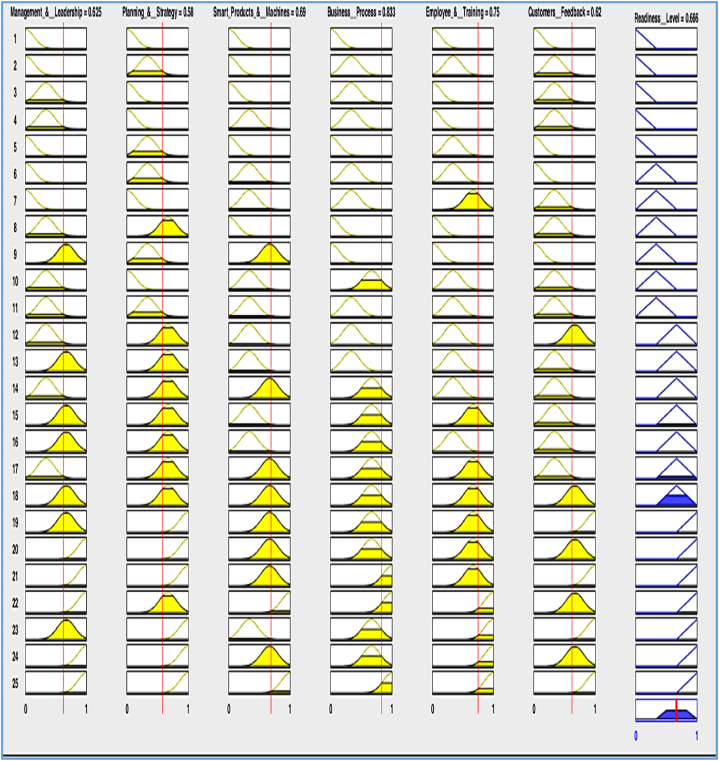
Readiness level of garments industry (Fuzzy model).

Then it is needed to determine the readiness of the garments industry according to the readiness model developed. For this, some modifications and calculations are needed. The answer to the questionnaire that the industry professionals gave was on a scale of four. Then all the question's answers were found on a scale of four. Then the mean score is plotted in a radar chart. To implement the model formulated in the fuzzy logic toolbox of MATLAB, the results are converted on a scale of one. Then all the data can be given as input in the fuzzy model. Fig. 9 on next page represents the result of the readiness of the garments industry in the fuzzy model.

So, it is seen that the case studied ABC garments industry shows a readiness level of 0.66.

The readiness level that is obtained from the studied garments industry is 0.66. That means that the garments industry has passed the novice stage and completely and now is in a stable "Survival" stage. From the radar chart and Fig. 9 is clear that if the garments industry can increase their planning and management dimensions to some extent, they soon can be in the "Professionals" readiness state. Also, they lack the customer's feedback dimension, which can be further increased by taking some steps in the planning process. So, the vital element that prevents them from reaching the "Professionals" readiness state is the planning and strategy dimension.

The model described in this paper uses a combination of AHP and a Fuzzy inference system where expert rating gets a mathematical validation and through fuzzy logic if-then rules the work yields an understandable result for better interpretation and decision-making.

From the result, it is evident that the strength of this work lies in the decision-making support that the industrial personnel will obtain from the result of the developed model. As the model is based on AHP, expert ratings from Bangladesh play a significant role in prioritizing the dimension, and it helps all the industries across the country make better decisions. However, the limitation is that it is most suitable for developing countries but not for developed countries where Industry 4.0 is already in the maturity stage. Also, this model is appropriate for manufacturing industries and not service-related industries. So the suggestion for future research is to extend its scope across all the industries. Moreover, the proposed model works well for readiness determination but not well if the company is in maturity for Industry 4.0.

## 5 Conclusion

The main objective of this paper was to propose a readiness model of Industry 4.0, keeping Bangladesh's perspective in mind. Following a thorough examination and research of the literature, numerous prior maturity models have been reviewed, and ultimately, a readiness model is developed using fuzzy logic and AHP. The model is based on a questionnaire approach. The readiness model developed works appropriately in the case of the manufacturing industry and assembly industry. However, the maturity model can't predict the readiness level of a service industry or similar industry types. In the future, the dimension called "Society 4.0″ will include education, business, and all types of industry, and they all will be in the paradigm of "Industry 4.0". So, the scope of the readiness model may need to be enhanced for future purposes. In this fast-paced world where industrial development rapidly increases a country's reputation on the world map, all developed countries in the world are entering the paradigm of "Industry 4.0". For a developing country like Bangladesh, it needs to quickly grasp the idea and the concept of "Industry 4.0″ to keep pace with the developed countries. The readiness model presented in this research work will help industrial personnel check their current readiness level in the era of "Industry 4.0," and they will be one step ahead of their journey of becoming global leaders in their respective fields.

## Data availability statement

The data that has been used is confidential.

## Additional information

No additional information is available for this paper.

## CRediT authorship contribution statement

**S.M. Fahim Faisal:** Writing - review & editing, Writing - original draft, Visualization, Validation, Software, Resources, Methodology, Formal analysis, Data curation, Conceptualization. **Sajal Chandra Banik:** Writing - review & editing, Writing - original draft, Validation, Supervision, Methodology, Formal analysis, Conceptualization. **Pranta Sen Gupta:** Writing - review & editing, Visualization, Formal analysis, Data curation.

## Declaration of competing interest

The authors declare that they have no known competing financial interests or personal relationships that could have appeared to influence the work reported in this paper.
